# Medical College Notes

**Published:** 1895-02

**Authors:** 


### Medical College Notes.

Dr. G. Frank Lydston began his third special course in regional surgery at the Masonic Hospital, Chicago, on Monday evening, January 7, 1895, to be continued during every Monday evening until completed. Subjects: Surgery of the Prostate, Bladder and Kidneys, including Calculus; Appendicitis; Surgery of the Rectum and Urethra; and Varicocele. The lectures will be illustrated by special charts and drawings and are free.

---

Dr. B. Mead Bolton, associate professor in bacteriology at Johns Hopkins University, delivered a lecture on Immunity, a Plea for the Establishment of Pasteur Institutes, at Alumni Hall, University Building, Friday evening, January 18, 1895, under the auspices of the faculty of Buffalo University Medical College. The medical profession of Buffalo was invited.

---

Dr. Virgil P. Gibney, of New York, surgeon-in-chief to the hospital for the ruptured and crippled, has been appointed professor of clinical surgery in the College of Physicians and Surgeons.

